# Cardiac Computed Tomography: State of the Art and Future Horizons

**DOI:** 10.3390/jcm11154429

**Published:** 2022-07-29

**Authors:** Gudrun M. Feuchtner, Fabian Plank, Christoph Beyer, Fabian Barbieri, Gerlig Widmann, Philipp Spitaler, Wolfgang Dichtl

**Affiliations:** 1 Department of Radiology, Innsbruck Medical University, 6020 Innsbruck, Austria; christoph.beyer@i-med.ac.at (C.B.); gerlig.widmann@i-med.ac.at (G.W.); 2 Department of Internal Medicine III, Cardiology, Innsbruck Medical University, 6020 Innsbruck, Austria; fabian.plank@i-med.ac.at (F.P.); philipp.spitaler@i-med.ac.at (P.S.); wolfgang.dichtl@tirol-kliniken.at (W.D.); 3 Department of Cardiology, Corporate Member of Freie Universität Berlin and Humboldt-Universität zu Berlin, Charité—Universitätsmedizin Berlin, Hindenburgdamm 30, 12203 Berlin, Germany; fabian.barbieri@charite.de

Cardiac computed tomography (CT) has evolved over the past 20 years from an alternative, promising noninvasive imaging modality to a Class I indication for the non-invasive evaluation of patients with low-to-intermediate, pre-test probability of coronary artery disease (CAD), as per the European Society of Cardiology (ESC) guidelines published in 2019 [1].

Most recently, the American Heart Association (AHA) Chest Pain Guidelines 2021 further strengthened the position of cardiac CT in the clinical management of patients with suspected CAD and recommended coronary computed tomography angiography (CTA) as first-line imaging modality [2].

The advantage of cardiac CT lies in the comprehensive evaluation of coronary arteries and all other cardiac structures, such as cardiac chambers and valves. As such, CTA offers high-resolution imaging of coronary arteries, plaque morphology, stenosis severity and through using advanced post-processing 3D/4D visualization and flow modeling (CT FFR) [3]. Modern technology has created a wide potential for CT, including AI-assisted quantitative image analysis, as well as dual-energy and, more recently, multi-spectral CT imaging.

The rise of CT in cardiovascular applications was initiated with the introduction of coronary artery calcium scoring (CACS) by electron beam CT in 1990. Arthur Agatston first described a method for the quantification of coronary calcium scoring based on volume and lesion density. In recent decades, a calcium score of zero (0) has shown an excellent negative predictive value for ruling out coronary artery disease (“the #powerofCACS0”) over a period of 10 years [4], as well as a strong predictive power for cardiovascular (CV) risk stratification outperforming a conventional risk factor score. Coronary artery calcium scoring (CACS) is currently recommended as a screening tool for low-to-intermediate asymptomatic individuals, and can be used as a baseline tool for a baseline cardiac check-up [4]. The Coronary Artery Calcium Data and Reporting System (CAC-DRS) provides a standardized classification on a per-patient basis, representing the total calcium score and the number of involved arteries in order to guide further management of patients with different degrees of calcified plaque burden. In contrast, coronary CTA, which includes the application of iodine contrast and a higher radiation dose, should be applied in symptomatic patients with a higher a priori pre-test probability for obstructive coronary artery disease. The major advantages of coronary CTA over CACS are the quantification of stenosis and qualitative evaluation of plaque morphology. Using the standardized Coronary Artery Calcium Data and Reporting System (CAC-DRS), additional prognostic discrimination for future coronary heart disease events can be provided [5].

The indications for coronary CTA mainly encompass patients with chest pain (atypical and typical) and a low-to-intermediate pre-test probability of CAD after a baseline physical check-up, including CV risk factor screening and/or other pre-testing.. For example, a patient without chest pain, but suspicious finding on another prior test would also qualify for coronary CTA—in order to detect or exclude obstructive CAD. 

In this Special Issue, we highlight the original research by Senoner et al. [6] regarding gender differences in patients with CACS 0 and ultralow CACS 0.1–0.9AU. In 1451 patients referred to cardiac CT for clinical indications, nonobstructive CAD (25.9% vs. 16.2%; *p* < 0.001), total plaque burden (2.2 vs. 1.4; *p* < 0.001), and HRP were found more often in males (*p* < 0.001), while the overall percentage of atherosclerosis was low (20.3% for females vs. 32.1% for males). Females were more often symptomatic for chest pain and overall, the event rate was very low. Of note, this cohort consisted of individuals in whom a previous baseline cardiological exam had already raised the suspicion for obstructive CAD, due to typical or atypical chest pain, high CV risk profile and/or other pathological findings of prior testing such as an ECG treadmill stress test or 24 h Holter. 

While the general strength of CACS 0 for excluding CAD has been proven by large cohort studies, there is an ongoing debate among the scientific community regarding the strength of CACS 0 in certain individuals, for example high-risk symptomatic persons, younger patients < 40 years with a high-risk profile or diabetics, or those with a high genetic predisposition such as familiar hypercholesterinemia (FH), who carry a higher risk to develop non-calcified plaque at a younger age. Currently, coronary CTA is the only non-invasive diagnostic tool, which allows for the detection of such lesions (especially, in the presence of CACS 0).

Most studies have shown that the prevalence of non-calcified plaque in CACS 0 patients is low, and especially, the rate of obstructive disease is very low [7] but depends other factors such as on age and gender [8].

Mortensen et al. [8] provided the scientific evidence most recently in a large cohort study of 23 759 persons in the Danish population, that the presence of non-calcified plaque in CACS 0 patient is indeed much higher in younger individuals < 40 years [8]. Overall, the prevalence of obstructive CAD was relatively low across all age groups, ranging from 3% (in those younger than 40 years) to 8% in patients older than 70 years. In patients with obstructive CAD, only 14% had a CAC score of 0. However, the prevalence declined linearly across age groups from 58% (younger than 40 years, to 34% (aged 40 to 49 years), 18% (50 to 59 years) and only 5% (52 of 964) among those who were 70 years or older. The added diagnostic value of a CAC score of 0 significantly decreased at a younger age [8].

This is especially important as low attenuation plaques (LAP < 30HU) are strong predictors for CV events, with a higher incremental value than the coronary calcium score, and have an independent prognostic value, as shown by numerous single-centric studies [9,10,11] and a prospective multicentric study by Williams et al. [12] (SCOT HEART) or the multicentric ICONIC trial [13].

A low-attenuation plaque (LAP) indicates a lipid-rich necrotic core (“vulnerable”) lesion, at risk to rupture and causing major adverse cardiovascular events. Therefore, the risk stratification of a patient is improved by adding LAP as a “high-risk-plaque (HRP)” criterion. This has been recently discussed regarding the implementation into LDL management—in such patients, a lower c-LDL value should be targeted, in order to improve CV outcomes. Of note, the presence of vulnerable “high-risk” plaque is also associated with a higher probability of ischemia, even at a lower degree of stenosis (such as intermediate, 50% stenotic lesions) [14].

Importantly, out of the wide array of CV risk factors, smoking and obesity have the strongest association with a “high-risk” plaque [15], but also diabetes. Therefore, standardized CTA reporting guidelines do recommend to add the presence of a vulnerable plaque (label “V”), in addition to stenosis severity, which is commonly classified as minimal (<25%), mild 25–49%, moderate (50–69%) and severe (70–99%) in the CAD-RADS classification [16]. While the detection of a high-risk plaque using a visual or semi-quantitative tool is feasible, it is time-consuming and cumbersome in clinical practice. Newly introduced AI-assisted plaque analysis tools [17] offer the advantage of a fully automated plaque analysis, including plaque characteristics and total plaque burden, and the percent of atheroma burden. 

On the other hand, most recently, in a large Danish cohort study (23,143 patients), it was shown that even in high-risk patients with high c-LDL, the combination of coronary CTA and CACS (including both noncalcified and calcified plaque evaluation) provided a high negative predictive value (NPV) to ensure favorable CV outcomes [18].

Beyond coronary arteries, cardiac CT offers a detailed morphological evaluation of cardiac valves and other structural heart diseases. Despite the fact that cardiac CT is used for preprocedural planning of many transcutaneous interventions (e.g., transcatheter aortic valve implantation (TAVI), left atrial appendage occlusion, transcatheter mitral valve replacement, etc.), one should not forget to evaluate coronary arteries with the exact same assessment. In a retrospective case–control study published in this Special Issue, the relationship of bicuspid valve morphology and the severity of CAD was evaluated in patients with aortic stenosis. Interestingly, patients with bicuspid valves had a lower CAD burden and severity of coronary calcium, as compared to patients with tricuspid valves [19]. This could be due to a genetic predisposition, or flow-mediated. 

In conclusion, cardiac CT has evolved into a reliable imaging modality in clinical practice. Whilst its main application in practice is the non-invasive evaluation of coronary artery disease, cardiac CT has gained a valuable position in the context of structural heart disease integrated into a multimodality work-up. Cardiac CT awaits a prosperous future: Artificial Intelligence (AI)-assisted tools allow for fully automated quantitative image analysis, and enhance the accuracy and efficacy in daily practice. Furthermore, novel CT technology, such as the recently introduced photon-counting CT [20], allow for high-resolution and multispectral energy imaging at a very-low-radiation dose, creating further diagnostic benefits, and the potential for a reduction in diagnostic invasive coronary angiography procedures [21].
